# Outsmarting Nightmare: Drug Resistance in *Acinetobacter baumannii*

**DOI:** 10.1155/cjid/9591804

**Published:** 2025-12-29

**Authors:** Annepu Praveen, Amjuri Sinduja, Mukesh Kumar Yadav, Divakar Sharma, Amit Singh

**Affiliations:** ^1^ Infectious Disease and Translational Research Lab, Department of Microbiology, Central University of Punjab, Bathinda, Punjab, India, cup.ac.in; ^2^ Department of Biotechnology, Graphic Era (Deemed to be) University, Dehradun, 248002, India

**Keywords:** *Acinetobacter baumannii*, AgNPs, antiphage resistance, beta-lactamases, CNPs, CRAB, drug resistance, efflux pumps, MDR, NEPT

## Abstract

*Acinetobacter baumannii* is a critical nosocomial pathogen recognized by the WHO as a top‐priority threat due to its extensive drug resistance, particularly in carbapenem‐resistant *Acinetobacter baumannii* (CRAB) strains. According to recent CDC reports, CRAB is one of the most urgent causes of hospital‐acquired infections, driving longer hospital stays, higher costs, and mortality. This pathogen employs multiple resistance mechanisms, including efflux pumps, porins like OmpA, making it highly difficult to treat. Resistance is further shaped by β‐lactamases, integrons, and mobile genetic elements. The emergence of MDR, XDR, and PDR strains signals the looming postantibiotic era, where conventional therapies are increasingly ineffective. While several reviews have provided in‐depth analyses of individual mechanisms and some comprehensive overviews, this work provides a broad overview of resistance strategies and genetic determinants, including *tet(A/B)*, *cmlA*, and *mdfA*. Importantly, we also highlight emerging alternatives such as novel emergent phage therapy (NEPT), which proactively counters antiphage resistance and nanoparticles (CNPs), which resensitize resistant isolates and enhance antibiotic efficacy. By combining mechanistic insights with emerging interventions, this review provides a comprehensive perspective on CRAB resistance and highlights research priorities for mitigating its global threat.

## 1. Introduction

Antimicrobial resistance (AMR) is a growing health crisis, burdening healthcare systems with longer hospitalization, higher costs, and increased mortality. Despite the development of new antimicrobials, resistance continues to spread, with nearly 5 million deaths worldwide linked to resistant infections in 2019 [1] and projections of up to 10 million annual deaths by 2050 [1–3]. Among the most concerning pathogens is *Acinetobacter baumannii*, a Gram‐negative opportunist and a member of the ESKAPE group, notorious for its ability to “escape” antimicrobial action. It causes a wide spectrum of nosocomial infections—from ventilator‐associated pneumonia to bloodstream, urinary tract, and wound infections—particularly in immunocompromised patients. The World Health Organization has identified carbapenem‐resistant *A. baumannii* (CRAB) as a critical priority pathogen [4]. With the outbreak reported worldwide, especially in the East Mediterranean and Asia‐Pacific regions [5], although several reviews have examined *A. baumannii* resistance, which deeply focused on individual mechanisms (e.g., β‐lactamases, efflux pump, or polymyxin resistance) or specific drug classes. However, significant taxonomic changes have occurred within the *Acinetobacter* genus, with *A. baumannii* over the last 30 years [6]. The emergence of strains resistant to all known antibiotics underscores the urgent need for global action [7, 8]. By contrast, this review provides a broad, comprehensive overview across all known resistance mechanisms and drug categories, integrating molecular insights with clinical consequences. In the emerging postantibiotic era, where treatment options are severely limited, innovative alternatives are being explored. Novel strategies such as the development of vaccines against AMR, and phage therapy, particularly the novel emergent phage therapy (NEPT) approach, have shown clinical promise by using a preoptimized multiphage cocktail to reduce CRAB burden and delay resistance development [9, 10]. However, bacterial antiphage resistance remains a key obstacle [11, 12]. A combination of nanoparticles with phage is a very effective approach to tackle this [13, 14]. In parallel, whole‐genome sequencing (WGS) provides unprecedented insights into the genetic basis of resistance [15]. It also highlights knowledge gaps, such as the role of outer membrane proteins (OMPs) in permeability, the dynamics of horizontal gene transfer, antiphage resistance, and unresolved mechanisms of polymyxin resistance that demand further study. By combining breadth with depth, this article not only summarizes the current state of resistance in *A. baumannii* but also outlines research priorities and clinical strategies, ranging from antimicrobial stewardship to emerging interventions like NEPT and nanoparticle‐based therapies to counter its rising threat.

### 1.1. What Are MDR, XDR, and PDR

1. MDR: The isolate shows resistance to a minimum of one agent or ≥ 3 of the following antimicrobial categories refers to the multidrug resistance.
  I. Antipseudomonal fluoroquinolones (ciprofloxacin or levofloxacin).
  II. Aminoglycosides (gentamicin, tobramycin, amikacin, or netilmicin).
  III. Ampicillin/sulbactam.
  IV. Antipseudomonal carbapenems (imipenem, meropenem, or doripenem).
  V. Extended‐spectrum cephalosporins (cefotaxime, ceftriaxone, ceftazidime, or cefepime).
2. XDR: Nonsusceptibility to all but ≤ 2 antimicrobial categories, excluding polymyxins and tigecycline.
3. PDR: Pan‐drug resistance refers to isolates that show resistance to all agents across all antimicrobial categories, including last‐resort drugs like polymyxins and tigecycline.

Intrinsic resistance, such as *A. baumannii’s* natural resistance to cephalosporins and penicillin, must be considered when defining MDR, XDR, and PDR [16, 17].

### 1.2. Mechanisms of Drug Resistance in *A. baumannii*

**Figure 1:**
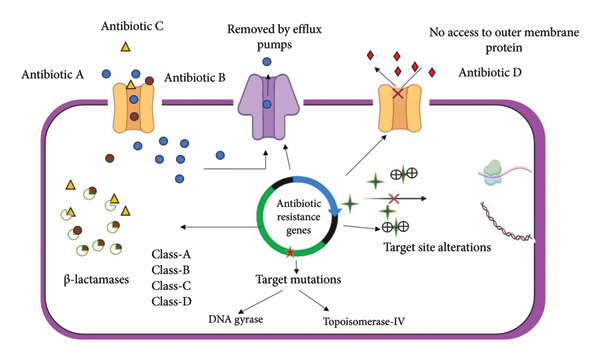
Diagrammatic representation of the drug resistance mechanism involved in *A. baumannii*.

The three major resistance mechanism groups provide the structure for our investigation (Figure 1 and Table 1):

1. Reduce the entry of drugs into the bacterial target site.
2. Enzymes inactivate antibiotics.
3. Alteration of the targets or cellular function due to mutations [18].

**Table 1:** Summarization of the potential targets and the mechanisms of drug resistance.

S. no	Resistance mechanism	Family/type	Pumps/gene/enzymes	Antibiotic effected
1.	Efflux pumps	ABC transporters	(Rare in Gram‐negatives)	Broad substrate, ATP‐driven export
		MFS (major facilitator superfamily)	*tet(A), tet(B), cmlA, mdfA*	Tetracyclines, minocyclines, chloramphenicol, ciprofloxacin other antibiotics
		RND (resistance‐nodulation‐division)	*AdeABC, AdeIJK, AdeFGH,* regulated by *AdeRS*	Aminoglycosides, tetracyclines, macrolides, fluoroquinolones, lincosamides
		MATE (multidrug and toxic compound extrusion)	*abeM, NorM-like*	Fluoroquinolones(ciprofloxacin, ofloxacin, norfloxacin), gentamicin, macrolides, chloramphenicol
		SMR (small multidrug resistance)	*abeS*	Small antimicrobial agents, dyes, and detergents

2.	Porins	Outer membrane proteins	*OmpA, HMP-AB, CarO*	Reduced entry, carbapenems(carO), broad resistance

3.	β‐lactamases	Class A (ESBLs)	*SHV, TEM, KPC, CTX-M, PC1* Genes: *blaTEM, blaSHV, blaGES-11, blaPER-1-7, and blaVEB-1*	Degrade cephalosporins(aztreonam, ceftriaxone, cefotaxime, ceftazidime)
		Class B (MBLs)	*IMP, VIM, NDM, SIM*	Degrade most β‐lactams(except monobactams)
		Class C (ADCs)	*ADC-33, ADC-56* (ISAbal insertions)	Hydrolyze penicillins, ESCs(cefotaxime, ceftazidime)
		Class D (CHDLs)	*OXA-23, OXA-10, OXA-24, OXA-51, OXA-58, OXA-143, OXA-235*	Inactivates all β‐lactams, high affinity for carbapenems (OXA‐23, OXA‐51)

Mobile genetic elements (MGEs), such as integrons, transposons, and plasmids, facilitate resistance gene dissemination through horizontal gene transfer (HGT) via transformation, conjugation, and transduction.

#### 1.2.1. Reduce the Accessibility of Drugs to the Bacterial Target Site

The primary cause of drug resistance in bacteria is the reduced entry of drugs through OMPs due to the upregulation of efflux pumps and the downregulation of porins. In *A. baumannii*, drug entry is limited by the small size of its porins [19]. This is the first and major mechanism of drug resistance in bacteria.

##### 1.2.1.1. Efflux Pumps

The outer membrane restricts antimicrobials′ entry into Gram‐negative bacteria, while multidrug efflux pumps actively export various antimicrobials, contributing to multidrug resistance. These transporters, present in all living cells, help protect against harmful chemicals. Overexpression leads to decreased drug accumulation and increased minimum inhibitory concentration (MIC). Commonly expelled antimicrobials include macrolides, tetracyclines, and quinolones, and these efflux pumps can recognize a wide range of different substrates despite their specificity [20]. Five groups of efflux transporters have been identified in prokaryotes, with *A. baumannii* featuring transporters such as major facilitator superfamily (MFS), Tet(A), Tet(B), multidrug and toxic compound extrusion (MATE) AbeM, resistance‐nodulation‐division (RND) AdeABC, small multidrug resistance (SMR), and ATP binding cassette (ABC). These systems utilize proton motive force for energy, while ABC families require ATP hydrolysis to export substrates. Despite the antibiotic era, transporters that expel multiple antibiotics have not evolved significantly. Almost 10% of bacterial genes are linked to transportation, with many encoding efflux pumps [21, 22].

Tetracycline antibiotics, such as doxycycline and minocycline, are becoming less effective against *A. baumannii* due to the bacterium’s developing resistance mechanisms, particularly the increased activity of efflux pumps like AdeABC and AdeIJK [23, 24]. Tetracycline resistance in bacteria, particularly *A. baumannii*, is mainly driven by efflux pumps like TetA, TetB, and Tet39, which remove the antibiotics from cells. Ribosomal protection proteins also prevent tetracyclines from binding to the ribosome. Additionally, resistance genes on plasmids, such as tet(A), tet(B), and Tet(M), contribute to the widespread resistance seen in clinical isolates [25, 26]. Additionally, efflux pumps like AdeABC, AdeIJK, AdeFGH, and tetracycline‐specific pumps TetA and TetG help lower intracellular fluoroquinolone concentrations, contributing to resistance [27, 28]. The prevalence of these resistance mechanisms has significantly limited the use of fluoroquinolones in treating *A. baumannii* infections. Efflux pumps like Mef and Msr also contribute by expelling antibiotics from the cell. Macrolide antibiotics are limited in their effectiveness against *A. baumannii* infections. Resistance to lincosamide antibiotics like lincomycin and clindamycin can arise through ribosomal modification, efflux, and drug inactivation, with RND efflux pumps such as AdeABC, AdeFGH, and AdeIJK playing a key role [29, 30]. Mutations in the armA 16sRNA methylase gene and increased activity of RND efflux pumps (AdeABC) contribute to aminoglycoside resistance in *A. baumannii* [31]. Efflux pumps (RND pumps AdeABC) also contribute to resistance by ejecting the drug from the cell. Heteroresistance, where subpopulations show varying resistance levels, complicates *A. baumannii* infection treatments, highlighting the challenges in using polymyxins effectively against resistant strains [32, 33].

##### 1.2.1.2. ABC Transporter

ABC‐type efflux pumps are multidrug transporters that utilize ATP to expel antimicrobials from cells. Their architecture includes transmembrane domains (TMDs) and nucleotide‐binding domains (NBDs) that bind and hydrolyze ATP to facilitate transport [34]. ABC exporters are classified as heterodimeric or homodimeric, with the latter linked to antibiotic resistance in gram‐positive bacteria. Most homodimeric transporters have two identical nucleotide‐binding sites, whereas heterodimeric transporters feature a degenerate binding site that does not require ATP hydrolysis [35]. Members of this family rarely contribute to antibiotic resistance in Gram‐negative bacteria. Efflux pumps, such as drug—proton antiporters, use the proton motive force for antimicrobial expulsion. Key efflux pumps involved in multidrug resistance are from the RND, MFS, and SMR families [21].

##### 1.2.1.3. MFS Transporter

MFS is a group of secondary active transporters that travel from bacteria to humans [36]. These MFS‐affiliated efflux pumps exchange a proton for a tetracycline‐cation complex. One gene in Gram‐negative bacteria codes for an efflux protein, while another encodes a repressor protein [37]. Tetracycline resistance in *A. baumannii* is primarily mediated by the efflux pumps Tet(A) and Tet(B). Tet(A) provides resistance to tetracycline, while Tet(B) confers resistance to both tetracycline and minocycline. Glycylcyclines and novel tetracyclines are not affected by these pumps. These efflux pumps are specific to certain classes of antimicrobials and are usually found on transposons within conjugative plasmids. The tet(A) gene can spread horizontally among different genera of Gram‐negative bacteria [25, 37, 38]. CmlA and MdfA efflux pumps provide resistance to chloramphenicol, linked to the cmlA gene. Research suggested that *A. baumannii* strain AYE has this gene within an 86‐kb resistance island [39]. Several Enterobacteriaceae have a transporter called MdfA. An orthologue with 42.7% sequence similarity was recently identified in a clinical isolate of *A. baumannii*. Multidrug‐resistant bacteria expressing MdfA show resistance to chloramphenicol, ciprofloxacin, and other antibiotics.

##### 1.2.1.4. RND Transporter

The AdeABC pump of *A. baumannii* consists of AdeA, AdeB, and AdeC proteins and supports resistance to multiple antibiotic classes, including aminoglycosides. AdeB is part of the RND superfamily, which in gram‐negative bacteria forms complexes with membrane fusion proteins (MFPs) and outer membrane factors (OMFs). These tripartite units actively expel antibiotics. Recent studies have focused on the structures of these components. Additionally, the AdeRS two‐component system regulates the AdeABC pump in wild *A. baumannii* [40]. Members of this group of efflux pumps eliminate antimicrobial agents from bacterial cells using the proton motive force as an energy source. The AdeABC efflux pump system consists of AdeA (MFP), AdeB (multidrug transporter), and AdeC (OMP), which together enable the antimicrobial agent to cross both membranes. Overexpression of this system leads to resistance against various antibiotics, including erythromycin and tetracyclines, and is associated with decreased fluoroquinolone susceptibility. The genes for these components are located together in the genome, suggesting they form an operon, with the MFP and transporter genes typically co‐transcribed alongside their regulatory gene [41]. The AdeRS two‐component system, comprising the sensor kinase AdeS and response regulator AdeR, controls the expression of the efflux pump and transporter genes [40]. The sensor protein activates or deactivates this efflux pump in response to environmental cues. The pump can link to other OMPs like AdeK, making AdeC unnecessary for resistance. AdeK is part of a recently discovered efflux pump in *A. baumannii* that is being characterized.

##### 1.2.1.5. MATE Transporter

These bacterial transporters are widespread and can also be found in more advanced plants and animals. The transporters were initially identified as an agent that transports Na+ and cations in opposite directions, known as NorM, which was discovered in *Vibrio parahaemolyticus* [42]. The multidrug efflux pump AbeM, part of the MATE family, shows over 90% sequence homology and 70% identity with the NorM homolog from *A. baumannii* ATCC 19606. [43]. VcmA shows similarities to other proteins like PmpM, YdhE, and HmrH. The presence of this protein increases the MICs of norfloxacin, ofloxacin, ciprofloxacin, and gentamicin fourfold, and causes a twofold rise in MICs of trimethoprim, erythromycin, kanamycin, and chloramphenicol. It is linked to the sodium ion gradient and proton motive force associated with MATE family efflux pumps [44]. AbeM uses the proton motive force to drive antimicrobials out of the cell. Only a few nonspecific efflux pump inhibitors, such as reserpine and MC 207,110, have been studied for their effects on efflux pump overexpression in *A. baumannii* clinical isolates. Despite their nonspecificity, they help identify combined efflux effects. According to Ribera et al., the MIC of nalidixic acid decreased eightfold in 45% of unrelated *A. baumannii* isolates with MC 207,110, while in 33% of isolates, the MIC of ciprofloxacin fell at least fourfold with reserpine, without affecting nalidixic acid levels. This suggests the presence of multiple efflux pumps based on the varying MIC changes for these antibiotics [41, 45].

##### 1.2.1.6. SMR Transporter

SMR transporters are drug/metabolite transporters from the DMT superfamily, functioning as monomers and powered by the proton‐motive force. A study on AbeS, a putative drug efflux pump from a multidrug‐resistant strain of *A. baumannii*, investigated its role in AMR. When the abeS gene was expressed in hypersensitive *E. coli* KAM32, there was a reduced susceptibility to various dyes, antimicrobial agents, and detergents. Deleting abeS in *A. baumannii* confirmed its role in resistance to these compounds [46].

##### 1.2.1.7. Porins

Porins are OMPs that form channels, allowing hydrophilic solutes to pass through lipid bilayer membranes. They have multiple functions, including serving as targets for cell adhesion and binding bactericidal compounds in Gram‐negative bacteria. Bacteria can alter porin structures to evade antibacterial pressure or regulate expression in response to antibiotics, aiding their survival. Porins contribute to antibiotic resistance, particularly in *A. baumannii*, where the low‐permeability outer membrane protein A (OmpA, 40 kDa) plays a structural role and enhances resistance. Understanding porins′ functions is vital for developing new treatments for bacterial infections [47, 48]. OmpA’s involvement in AMR in *A. baumannii* is resistant to multiple antibiotics. OmpA is a key component of the outer membrane that protects the cell from external threats like antibiotics. Its exact role in AMR is not fully understood, still being investigated, but some studies have speculated that OmpA may help expel compounds from the periplasmic space, aiding in antibiotic resistance. Additionally, OmpA could interact with inner membrane efflux systems, enhancing the activity of efflux pumps that remove antibiotics from the cell, further increasing resistance. These proposed roles require further experimental validation to be definitively proven [49]. The primary OMP of *A. baumannii*, HMP‐AB, is a porin that allows solutes up to 800 Da to pass through, contributing to the bacterium’s antibiotic resistance. The diffusion rate of uncharged solutes through HMP‐AB channels is influenced by solute size. HMP‐AB’s penetration rates are significantly higher due to its abundance in protea liposomes, being about five times more than E. coli’s OmpF porin [50]. Recent investigations have focused on the carbapenem‐associated OMP (CarO, 29 kDa) in both carbapenem‐sensitive and resistant strains of *A. baumannii*. Studies suggest that CarO serves as a channel for L‐ornithine uptake and potentially for carbapenems, which could help in understanding antibiotic resistance in *A. baumannii* and in developing new treatment strategies [51].

## 2. Enzymatic Inactivation of Antibiotics

The main inactivating enzymes in drug resistance are β‐lactamases, which hydrolyze the β‐lactam drugs.

### 2.1. Beta‐Lactamases

**Figure 2:**
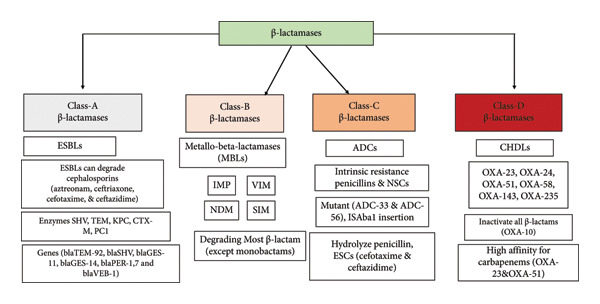
A comprehensive overview and functional diversity of β‐lactamase classes.

Beta‐lactamases are a class of enzymes that play a crucial role in the resistance of bacteria to antibiotics. These enzymes are responsible for breaking down beta‐lactam antibiotics, which are commonly used to treat bacterial infections. They are categorized into four different classes based on their unique sequence motifs and differences in how they break down the antibiotics. Class A, C, and D β‐lactamases (Figure 2) use an active site serine for opening of the β‐lactam ring, while the Class B enzymes are metallo‐β‐lactamases (MBLs) [52]. This classification system helps in understanding the various mechanisms of these enzymes and in developing strategies to combat antibiotic resistance (Table 2).

**Table 2:** The accounts of *A. baumannii* containing acquired metallo‐β‐lactamases.

Antibiotics	Gene	Sub‐gene	Location	Reference
(β‐lactams)	IMP	IMP‐1	Japan	[53]
		IMP‐2	Italy	[54]
		IMP‐4	China	[55]
		IMP‐5	Portugal	[56]
		IMP‐8	China	[57]
		IMP‐10	Japan	[58]^†^
	VIM	VIM‐1	Greece	[59]
		VIM‐2	Korea	[60]
		VIM‐3	Taiwan	[61]
		VIM‐4	Greece	[62]
		VIM‐11	Taiwan	[63]
	NDM	NDM‐1	India	[64]
	SIM	SIM‐1	Korea	[65]

*Note:* The table reports the first occurrence of a given gene in *A. baumannii* and the respective class of antibiotic resistance.

^†^GenBank accession number AB074436.

#### 2.1.1. Class A Beta‐Lactamases

Beta‐lactamases of Class A are enzymes that can cause resistance to various antibiotics. Penicillin, cephalosporins, monobactams, and carbapenems are a few of these. The activity range of these lactamases can be either narrow or broad. Point mutations are frequently the cause of the extended range. The majority of the time, narrow‐spectrum lactamases are only effective against penicillin. The range of antibiotics that the extended‐spectrum beta‐lactamases (ESBLs) can degrade is greater. Cephalosporins such as aztreonam, ceftriaxone, cefotaxime, and ceftazidime fall under this category [66]. Monitoring ESBL‐producing strains and their corresponding genes (*blaTEM-92*, *blaSHV*, *blaGES-11*, *blaGES-14*, *blaPER-1,7*, and *blaVEB-1*) is crucial in a medical context [67, 68]. Prominent class A enzymes include SHV, which has activity similar to TEM and was originally found on the chromosome of *K. pneumoniae*, as well as PC1, TEM, and CTX‐M, which are active against cefotaxime. KPC refers to *K. pneumoniae* carbapenemase [69]. Cephalosporin efficacy has been threatened by ESBL‐producing strains. Frequent monitoring of these genes is beneficial. A review of mutational adaptation in class A enzymes reveals critical insights into their acylation mechanism and broad activity against carbapenem substrates.

#### 2.1.2. Class B Beta‐Lactamases

MBLs are enzymes that contribute to multidrug resistance in bacteria by degrading almost all beta‐lactam antibiotics except monobactams. They are encoded by mobile DNA and require heavy metals, especially zinc, for their catalytic activity. They have four types of MBLs (IMP, VIM, NDM, and SIM) [70]. However, identifying MBL‐producing organisms can be challenging with traditional methods, making molecular techniques necessary for accurate detection. Recent research has shown that NDM lactamases do not negatively impact bacterial growth. Understanding the mechanisms of MBL production and detection is critical in the fight against multidrug‐resistant bacteria.

MBLs are categorized into three groups of subfamilies based on their active site structure and sequence diversity [70]. B1 enzymes are the most clinically significant due to their binuclear zinc center, with NDM and VIM being widely disseminated on MGEs. In contrast, subclasses B2 and B3 are typically found on the chromosomes of Gram‐negative bacteria. The ability to substitute zinc for other metal ions while retaining hydrolytic activity has driven research into the catalytic mechanisms of MBLs. However, the diversity within MBL subclasses complicates identifying common features in β‐lactam breakdown mechanisms [71, 72].

#### 2.1.3. Class C Beta‐Lactamases

*Acinetobacter* spp. possess natural resistance to penicillins and narrow‐spectrum cephalosporins due to the chromosomal class C beta‐lactamase, known as *Acinetobacter*‐derived cephalosporins (ADCs) [73, 74]. ADCs are naturally expressed at low levels and do not need induction like other class C β‐lactamases. The insertion of ISAba1 significantly boosts their expression, leading to resistance against extended‐spectrum cephalosporins like cefotaxime and ceftazidime [75]. ADC‐associated *bla* genes encode class C cephalosporinases, which hydrolyze particular antibiotics. Notably, ADCs do not have action against cefepime or carbapenem. Recent findings include mutant ADC compounds, such as ADC‐33 and ADC‐56, which widen the scope of hydrolysis to include cefepime, representing an evolving aspect of *Acinetobacter* resistance mechanisms [76].

#### 2.1.4. Class D Beta‐Lactamases

OXA enzymes, or oxacillinases, are a class of carbapenem‐hydrolyzing class‐D β‐lactamases (CHDLs) that can inactivate all β‐lactams, primarily from the OXA‐10 family, and are the main mechanism of carbapenem resistance, similar to class‐A and class‐C β‐lactamases [77]. Class‐D β‐lactamases, including blaOXA‐51, blaOXA‐23, blaOXA‐24, blaOXA‐58, blaOXA‐143, and blaOXA‐235, are serine‐dependent enzymes that have been transferred to plasmids, increasing their clinical threat [78]. Some OXA enzymes have a narrow substrate profile, accepting mainly penicillins and first‐generation cephalosporins, while others can also act on later‐generation cephalosporins and carbapenem antibiotics [79]. Single amino acid alterations in enzymes like OXA‐2 and OXA‐10 can expand their activity spectrum. Carbapenem resistance in *A. baumannii* is linked to the overexpression of OXA‐23 or OXA‐51 due to ISAba1 insertion in their promoters. Class‐D β‐lactamases effectively degrade penicillins and early‐generation cephalosporins, but not extended‐spectrum cephalosporins or aztreonam. Carbapenem hydrolysis is generally slow, with imipenem hydrolyzing faster than meropenem or doripenem (except for OXA‐2 and ‐10). Many OXA enzymes have a high affinity for carbapenems, often in the nanomolar range [80].

## 3. Alteration of the Target Sites by Mutations

Target site alterations can lead to antibiotic resistance in Gram‐negative bacilli. In *A. baumannii*, fluoroquinolone resistance is often due to mutations in *GyrA* and *ParC*, while imipenem resistance is linked to changes or overexpression of penicillin‐binding proteins (PBPs), particularly reduced *PBP1b* expression. Aminoglycoside resistance arises from modifying enzymes and 16S rRNA methyltransferases, causing target site methylation. Mutations in the QRDR of DNA gyrase and topoisomerase IV primarily drive fluoroquinolone resistance [81]. Fluoroquinolone resistance in *A. baumannii* mainly results from mutations in the QRDRs of the *gyrA* and *parC* genes. These genes encode DNA gyrase and topoisomerase IV, and mutations in them reduce fluoroquinolone effectiveness by hindering target binding, leading to resistance. A mutation changing Ser83 to Leu was found in the QRDR of *gyrA* in all *A. baumannii* isolates studied. Of the 30 isolates, mutations in *gyrB* included Glu479 to Asp (3.6%), Asp644 to Tyr (12.5%), and Ala677 to Val (37.5%). In *parC*, mutations were Ser80 to Leu (53.6%), Ser80 to Trp (3.6%), and Glu84 to Lys (37.5%) among 53 of 56 isolates (94.6%). In total, 27 (48.2%) fluoroquinolone‐resistant isolates had mutations in *gyrA*, *gyrB*, and *parC* [82].

Polymyxins, especially polymyxin B and colistin, are last‐resort treatments for multidrug‐resistant *A. baumannii* infections. Resistance to these antibiotics has emerged due to modifications in the bacterial outer membrane, primarily through the addition of phosphoethanolamine (pEtN) to the lipid A of lipopolysaccharides (LPS). This alteration reduces the bacteria’s negative charge, lowering polymyxin attraction. Various genes (intrinsic and plasmid encoded), *pmrA/pmrB*, *lpxA/lpxD*, *mcr1-10*, are often upregulated or mutated in resistant strains, which modifies the LPS [83]. To date, this mechanism of polymyxin resistance has been reported exclusively in *A. baumannii.* The loss of LPS, including its lipid A anchor, results from mutations in any of the initial three genes involved in lipid A biosynthesis: *lpxA*, *lpxC*, and *lpxD*. Examination of 21 independently derived colistin‐resistant mutants of *A. baumannii* strain ATCC 19606 revealed that each mutant harbored a distinct mutation in one of these three lipid A biosynthesis genes [84–86]. *A. baumannii’s* resistance to macrolide–lincosamide–streptogramin (MLS) antibiotics primarily stems from erm genes that modify the ribosome’s antibiotic‐binding site, rendering the drugs ineffective. These resistance mechanisms are often transferred through MGEs, increasing MLS resistance in *A. baumannii.* [87].

Glycopeptides like vancomycin are mainly effective against Gram‐positive bacteria, but certain strains of *A. baumannii*, which are Gram‐negative, have outer membrane structures that prevent glycopeptides from binding effectively. [88]. Fosfomycin resistance in *A. baumannii* is often due to inactivating enzymes like FosA and mutations in transport systems that reduce drug uptake, leading to lower accumulation in bacterial cells [89, 90]. Resistance to rifamycins in *A. baumannii* can arise from mutations in the rpoB gene, which encodes RNA polymerase’s beta subunit. These mutations reduce the effectiveness of rifamycins by preventing binding to their target [91].

### 3.1. HGT

Conjugation facilitates the transfer of antibiotic resistance genes in Gram‐negative bacteria. While many studies have explored this, few have documented the successful transfer of resistance genes with integrons from clinical *Acinetobacter* spp. to environmental isolates. Key resistance genes in *A. baumannii* include blaGES‐14, blaIMP, blaVIM, and blaSIM, with plasmid‐located genes corresponding to β‐lactam resistance (blaGES‐11), carbapenem resistance (blaIMP, blaVIM, blaOXA‐23, blaOXA‐24, blaOXA‐58, blaNDM‐1), sulfonamide (*sul2*), and streptomycin (*strAB*) [92, 93]. Transposable elements, such as transposons and insertion sequences, play a role in AMR in *A. baumannii*. Notable insertion sequences like ISAba1, ISAba2, ISAba3, ISAba4, and IS18 are associated with carbapenemase gene expression [94].

### 3.2. Transposons

Transposons, ranging from 3 to 40 kb, may contain multiple genes and are classified as composite transposons (with resistance genes bordered by insertion sequences) or complex transposons (with more intricate structures). Tn2006, Tn2007, and Tn2008 are linked to blaOXA‐23. In Tn2006, two identical ISAba1 insertion sequences flank the blaOXA‐23 gene in opposite directions [95].

### 3.3. Integrons

Integrons are natural cloning and expression systems that can incorporate ORFs by site‐specific recombination and convert them into functional genes through a promoter sequence [96]. Five different categories of mobile integrons have been identified so far, which are classified based on the encoded integrases. It is widely acknowledged that three of these classes (1, 2, and 3) play a crucial role in spreading AMR genes [97].

#### 3.3.1. Drugs Modifying Enzymes

Aminoglycosides like gentamicin, amikacin, and tobramycin are commonly used to treat *A. baumannii* infections. However, resistance to these antibiotics is rising, mainly due to aminoglycoside‐modifying enzymes like acetyltransferases, nucleotidyl transferases, and phosphotransferases. These enzymes, encoded by *aadB*, *aadA*, *apa6*, and *aacc1* genes, deactivate aminoglycosides by modifying the drug, preventing its binding to bacterial ribosomes [98].

*A. baumannii* develops resistance to sulfonamides mainly through alternative dihydropteroate synthase enzymes with lower affinity for these drugs. The presence of resistance genes like sul1 and sul2, which produce sulfonamide‐resistance enzymes, also plays a significant role [99].

#### 3.3.2. Current and Future Treatment Strategies Against Drug‐Resistant *A. baumannii*

##### 3.3.2.1. Combination Therapies

To tackle antibiotic resistance, currently, combination therapies are on the rise to target multiple bacterial pathways at once. Combining polymyxins (such as colistin) with carbapenems or tigecycline has an increased effect against CRAB. Polymyxins act on the outer membrane, which prevents the diffusion of carbapenems or tigecycline, enhancing bacterial eradication while reducing the development of resistance [100–102], evidencing their use in multidrug‐resistant *A. baumannii* infections, particularly in ventilator‐associated pneumonia and bloodstream infections. Sulbactam, a β‐lactamase inhibitor, possesses intrinsic bactericidal activity via the binding of PBPs. The success of combination therapies is strain‐dependent due to the resistance profile of the bacteria. Rifampicin combinations can yield mixed results; some studies show synergy, while others write off any additional benefit [4]. Hence, antimicrobial susceptibility testing‐based personalized therapy is crucial for patient outcomes.

##### 3.3.2.2. Novel β‐Lactamase Inhibitors

The emergence of β‐lactamase‐producing *A. baumannii*, particularly those with OXA‐type carbapenemases and MBLs, has resulted in the development of novel β‐lactamase inhibitors to improve the efficacy of β‐lactam antibiotics.

• Avibactam, a non‐β‐lactam β‐lactamase inhibitor, is effective against class A (KPC), class C (AmpC), and some class D (OXA‐48) β‐lactamases. The combination of ceftazidime and avibactam has shown efficacy against CRAB strains, but its use is limited due to the presence of MBLs, which are not inhibited by avibactam [78].
• Relebactam, when coupled with imipenem‐cilastatin, is effective against CRAB isolates that produce class A and C β‐lactamases. However, it is ineffective against MBLs, as is avibactam [4].

#### 3.3.3. Phage Therapy: A Novel Strategy for Intractable CRAB Infections

Phage treatment is showing promise as an alternative to standard antibiotics. Phage therapy has emerged as a compelling novel strategy for addressing pulmonary infections, a critical need highlighted during the COVID‐19 pandemic, where secondary infections pose a significant risk. A compassionate use study demonstrated the potential of Phage intervention by enrolling four critically ill COVID‐19 patients (aged 62–81 Years) with CRAB infections that had persisted for 6 to 50 days, defying multiple high‐grade antibiotics. To proactively counter resistance, the NEPT strategy was key. Initial susceptibility testing identified the podoviral phage ɸAb124 as the only effective lytic agent. NEPT involved using ɸAb124 to select for resistant strains in vitro, allowing the identification of a potent “second line” Phage. This second Phage, ɸAb121 (Myoviridae), was chosen due to its significant morphological and genetic differences from ɸAb124 and its confirmed in vitro synergistic activity, thereby protecting the treatment from rapid resistance development. Treatment with this preoptimized two‐phage cocktail at two successive 10^−9^ PFU doses was associated with a favorable outcome, leading to a reduction in CRAB burden in all patients [12], found that a combination of three phages successfully eliminated CRAB biofilms in a mouse model of pneumonia. Synthetic biology advancements have made designing phages with improved lytic activity and resistance mechanisms possible. For example, phages designed to carry CRISPR‐Cas9 systems can selectively destroy resistance genes in *A. baumannii* [43]. Bacterial anti‐phage resistance is a major barrier to phage therapy. In Wu et al.’s [12] study, despite demonstrated in vitro synergy between phages ɸAb124 and ɸAb121, resistance developed in four of six treatments. And *A. baumannii* isolate TP3, collected 8 days after phage therapy, showed resistance to both initial phage cocktails(ΦPC and ΦIV) [11, 12]. Interestingly, this resistance was sometimes linked to reduced virulence, where prior antibiotic treatment had failed. To address such limitations, combining phages with nanoparticles offers targeted delivery, controlled release, and improved stability, enhancing therapeutic outcomes against multidrug‐resistant *A. baumannii* [13, 14].

#### 3.3.4. Nanoparticle‐Based Therapies

Silver nanoparticles (AgNPs) are a promising option for treating CRAB infections, showing strong antimicrobial activity with an MIC of 2.5 µg/mL. This study assessed the potential of AgNPs in combination with selected antibiotics. In vitro results revealed a synergistic effect between AgNPs and both polymyxin B, PMP and Rifampicin, while Tigecycline showed only an additive effect. In a CRAB‐infected mouse model, a low‐dose combination of AgNPs (2 mg/kg) and PMB (10 µg/kg) achieved a 60% survival rate, significantly better than either drug alone, while also reducing bacterial colonization in key organs. This highlights AgNPs’ potential to enhance antibiotic efficacy against resistant strains [103]. Chitosan nanoparticles (CNPs) also provide a promising strategy against MDR *A. baumannii*. The spherical nanoparticles, characterized by a positive charge (+37.7 mV), exhibit inherent antimicrobial activity but are primarily valuable as an adjuvant to enhance conventional drug efficacy. Crucially, studies show that CNPs successfully resensitize highly resistant clinical isolates to key antibiotics 100% of isolates were resensitized to meropenem, and 88.8% to colistin. This demonstrates the CNPs’ potential to overcome resistance mechanisms by boosting the power of both single‐ and two‐drug regimens [104]. WGS has emerged as a powerful tool for understanding antibiotic resistance, offering high‐resolution insights into infection and facilitating precision medicine. In the case of pan‐drug‐resistant *A. baumannii,* WGS has revealed the genetic underpinnings of resistance and virulence. In Wang et al.’s study, MDR‐SHHO2 strain analysis identified 12 genes encoding aminoglycoside‐modifying enzyme (e.g. aac(3)‐la, aph(3′)‐vla, and ant(2″)‐la etc) as well as 45 pathogenicity islands that increases virulence potential. Integrating WGS into clinical practices enables healthcare providers to tailor treatment to resistance profiles and simultaneously directs researchers toward new drug target and monitoring strategies for emerging resistant strains [15, 105].

## 4. Conclusion

CRAB exemplifies the urgent global threat of AMR, where MDR, XDR, and PDR strains severely limit therapeutic choices. Despite extensive research, critical knowledge gaps persist, including the role of OMPs in permeability, the mechanisms of polymyxin resistance, HGT, biofilm‐mediated persistence, and the distinction between intrinsic and acquired resistance. Addressing these gaps is vital for identifying new drug targets and guiding innovative therapies. Emerging strategies such as phage therapy, nanoparticle‐based interventions, and the integration of WGS offer promising avenues for precision medicine, enabling tailored treatments while revealing novel resistance determinants like aminoglycoside‐modifying enzymes like aac(3), and pathogenicity islands. Clinically, urgent priorities include strengthening antimicrobial stewardship, implementing rapid diagnostics, and refining combination therapies. Moving forward, global efforts must focus on translating these scientific advances into accessible clinical solutions, ensuring both the preservation of existing antibiotics and the development of effective next‐generation interventions.

## Ethics Statement

The authors have nothing to report.

## Consent

The authors have nothing to report.

## Conflicts of Interest

The authors declare no conflicts of interest.

## Author Contributions

Annepu Praveen, Amjuri Sinduja, Mukesh Kumar Yadav, and Amit Singh wrote the initial draft and prepared the figures. Amit Singh and Divakar Sharma edited the revision and supervised. All authors reviewed the final manuscript.

## Funding

The authors received no specific funding for this work.

## Data Availability

The authors have nothing to report.
